# Right upper lobectomy via selective right upper lobe isolation using a left-sided double-lumen tube placed in the right bronchus intermedius: a case report

**DOI:** 10.1186/s12893-026-04259-3

**Published:** 2026-10-03

**Authors:** Shengwen Song, Lingli Ding, Han Xu

**Affiliations:** 1 Department of Anesthesiology, The First Affiliated Hospital, Zhejiang University School of Medicine, 1367 Wenyi West Road, Hangzhou, Zhejiang 311121 China; 2 Department of Nursing, The First Affiliated Hospital, Zhejiang University School of Medicine, Hangzhou, 311121 Zhejiang China

**Keywords:** Double-lumen tube, Lung isolation, Right upper lobectomy, Diaphragmatic hernia, One-lung ventilation

## Abstract

**Background:**

One-lung ventilation (OLV) using a left-sided double-lumen tube (DLT) in the left main bronchus is standard for right thoracic surgery. When the contralateral left lung is severely compromised, this approach forces OLV to rely on the dysfunctional lung and risks critical hypoxemia.

**Case presentation:**

An 84-year-old woman with a left diaphragmatic hernia causing approximately 70% compression and atelectasis of the left lung was scheduled for right upper lobectomy. Preoperative assessment indicated that standard DLT placement would necessitate complete right lung collapse, placing the patient at risk of life-threatening hypoxemia. A 32-Fr left-sided DLT was intentionally inserted into the right bronchus intermedius under fiberoptic bronchoscopy (FOB). The bronchial cuff was positioned at the orifice of the right upper lobe (RUL) bronchus to achieve selective occlusion. Ventilation was delivered through the tracheal lumen to the left lung and through the bronchial lumen to the right middle and lower lobes. The RUL collapsed completely. Intraoperative oxygenation remained stable, with SpO_2_ greater than 95%. The patient underwent an uneventful thoracoscopic right upper lobectomy, was extubated immediately after surgery, and was discharged on postoperative day 6.

**Conclusions:**

Intentional placement of a left-sided DLT into the right bronchus intermedius is a feasible strategy for selective RUL isolation when standard OLV is contraindicated due to severe contralateral lung compromise. Preoperative computed tomography (CT)-based airway assessment and fiberoptic bronchoscopic guidance are recommended for safe implementation.

## Background

Lung isolation is essential for many thoracic surgical procedures [1]. Left-sided double-lumen tube (DLT) is preferred over right-sided tube because it offers easier intubation, more reliable positioning, and effective bilateral suctioning. For right lung surgery, a left-sided DLT in the left main bronchus is the preferred choice [2].

This standard approach enables complete collapse of the right lung while ventilating the left. However, this approach becomes untenable when the left lung is severely compromised [3]. Conditions such as diaphragmatic hernia with compressive atelectasis can render the left lung dysfunctional [4]. In these patients, conventional DLT placement forces one-lung ventilation (OLV) to depend exclusively on an already dysfunctional lung, which may lead to rapid hypoxemia [5].

We report a case of right upper lobectomy in a patient with a left diaphragmatic hernia causing significant left lung collapse. Intentional insertion of a left-sided DLT into the right bronchus intermedius allowed selective occlusion of the right upper lobe (RUL) orifice, preserving ventilation to the right middle and lower lobes while the compromised left lung continued receiving ventilation through the tracheal lumen.

## Case presentation

**Fig. 1:**
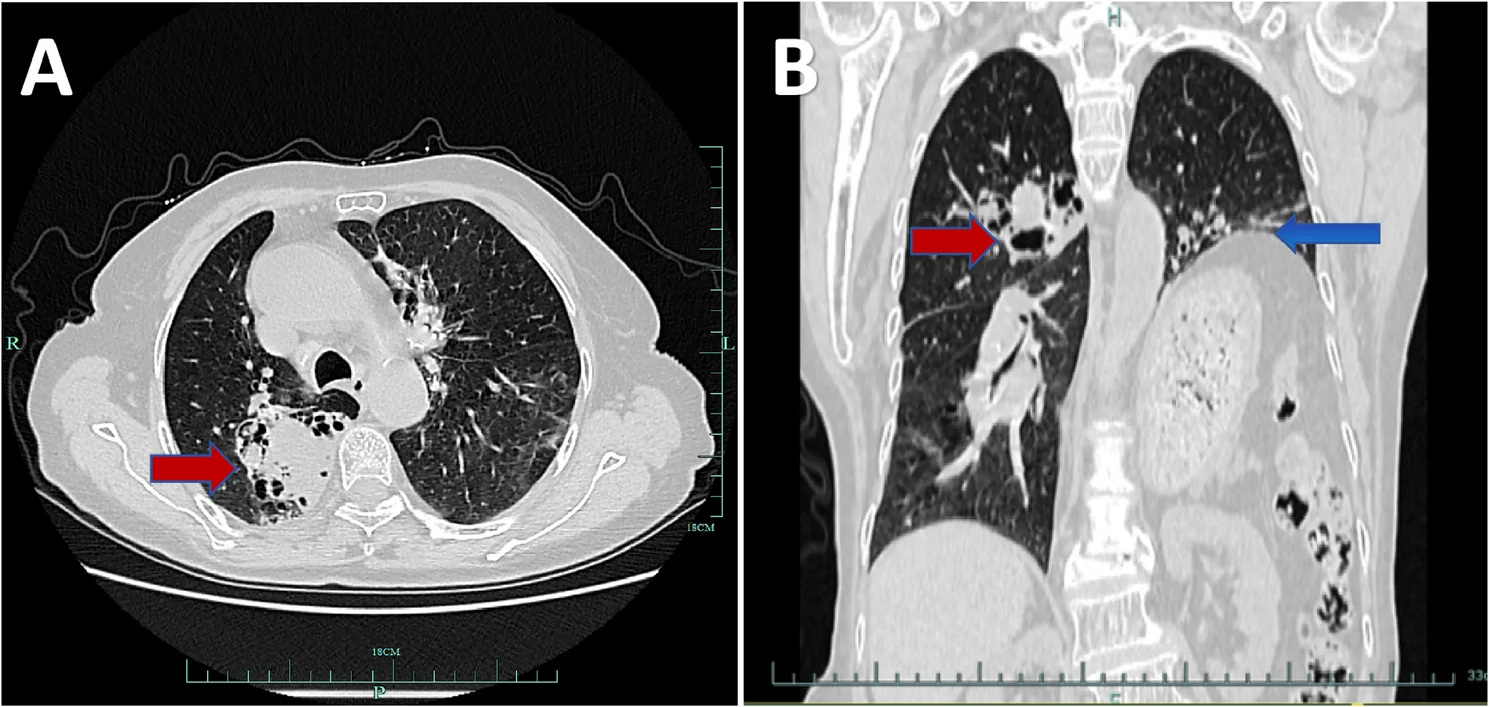
Preoperative chest computed tomography. **A** Axial image showing a thick-walled cavitary lesion (approximately 5.5 × 6.0 cm) in the right upper lobe (red arrow), with surrounding parenchymal destruction and bronchiectatic changes. **B** Coronal reconstruction showing the right upper lobe cavity (red arrow) and abdominal contents herniating into the left hemithorax (blue arrow), causing approximately 70% compression of the left lung

An 84-year-old woman (height 157 cm, weight 49 kg, body mass index 19.9 kg/m²) presented with a pulmonary fungal infection. She had experienced chronic cough with hemoptysis for seven months before admission. The hemoptysis was intermittent, presenting blood-streaked sputum several times a week, with each episode estimated at less than 20 mL and no massive bleeding. Chest computed tomography (CT) showed an RUL cavitary lesion (approximately 5.5 × 6.0 cm) with an irregular thick wall, containing intracavitary necrotic debris and a peripheral air gap, accompanied by parenchymal destruction, bronchiectasis, and cystic spaces. Additionally, CT revealed a left diaphragmatic hernia with approximately 70% compression and atelectasis of the left lung (Fig. 1). Microbiological confirmation was not obtained preoperatively. A working diagnosis of pulmonary fungal infection was made on the basis of the characteristic CT appearance (a thick-walled cavity containing an intracavitary mass with an air crescent sign) and was subsequently confirmed by histopathological examination of the resected specimen. She received voriconazole 200 mg twice daily for seven months; however, the lesion showed no significant radiographic improvement, and hemoptysis persisted.

Pulmonary function testing showed a forced vital capacity (FVC) of 1.50 L, a forced expiratory volume in 1 s (FEV_1_) of 1.11 L (68.3% predicted), and an FEV_1_/FVC ratio of 73.9%. The diffusing capacity of the lung for carbon monoxide (DLCO) was 53.9% predicted. Predicted postoperative pulmonary function was estimated using a non-validated functional approximation based on segment-counting: because the left lung was compressed by approximately 70% on CT, each of the nine left-sided bronchopulmonary segments was assigned a 30% functional weight as a pragmatic surrogate for regional contribution. This yielded 12.7 preoperative functional segment equivalents (10 right-sided segments × 100% + 9 left-sided segments × 30%), of which 9.7 would remain after resection of the three RUL segments. Accordingly, predicted postoperative FEV₁ (ppoFEV₁) was estimated at 52.0% (68.3% × 9.7/12.7 ≈ 52.0%) and predicted postoperative DLCO (ppoDLCO) at approximately 41.2% (53.9% × 9.7/12.7 ≈ 41.2%). She required no supplemental oxygen, with a resting SpO_2_ of 95% on room air. Eastern Cooperative Oncology Group Performance Status (ECOG) was 1, and she could climb 2 flights of stairs without stopping, corresponding to more than 4 metabolic equivalents.

After multidisciplinary team discussion among thoracic surgeons, pulmonologists, and anesthesiologists, surgical resection was indicated on the basis of persistent hemoptysis refractory to medical therapy, the risk of life-threatening hemorrhage from the cavitary lesion, and disease radiologically confined to the RUL with acceptable predicted postoperative lung function. This decision was consistent with current guideline recommendations for selected patients with localized pulmonary aspergillosis unresponsive to medical therapy [6, 7]. A video-assisted thoracoscopic surgery (VATS) right upper lobectomy was planned.

Physical examination showed the patient was afebrile with stable vital signs. Decreased breath sounds were noted over the left lower hemithorax. Preoperative arterial blood gas (ABG) analysis while breathing room air showed pH 7.44, PaO₂ 75.0 mmHg, and PaCO₂ 40.3 mmHg. The primary anesthetic challenge was to achieve reliable collapse of the RUL while maintaining adequate gas exchange, as the left lung could not support effective OLV.

In the operating room, standard monitoring, including invasive arterial pressure monitoring, was established. Rapid sequence induction was performed because of aspiration risk, using midazolam 2 mg, etomidate 10 mg, sufentanil 20 µg, and rocuronium 50 mg. Anesthesia was maintained with propofol (250–350 mg/h) and remifentanil (0.5-1.0 mg/h).

**Fig. 2:**
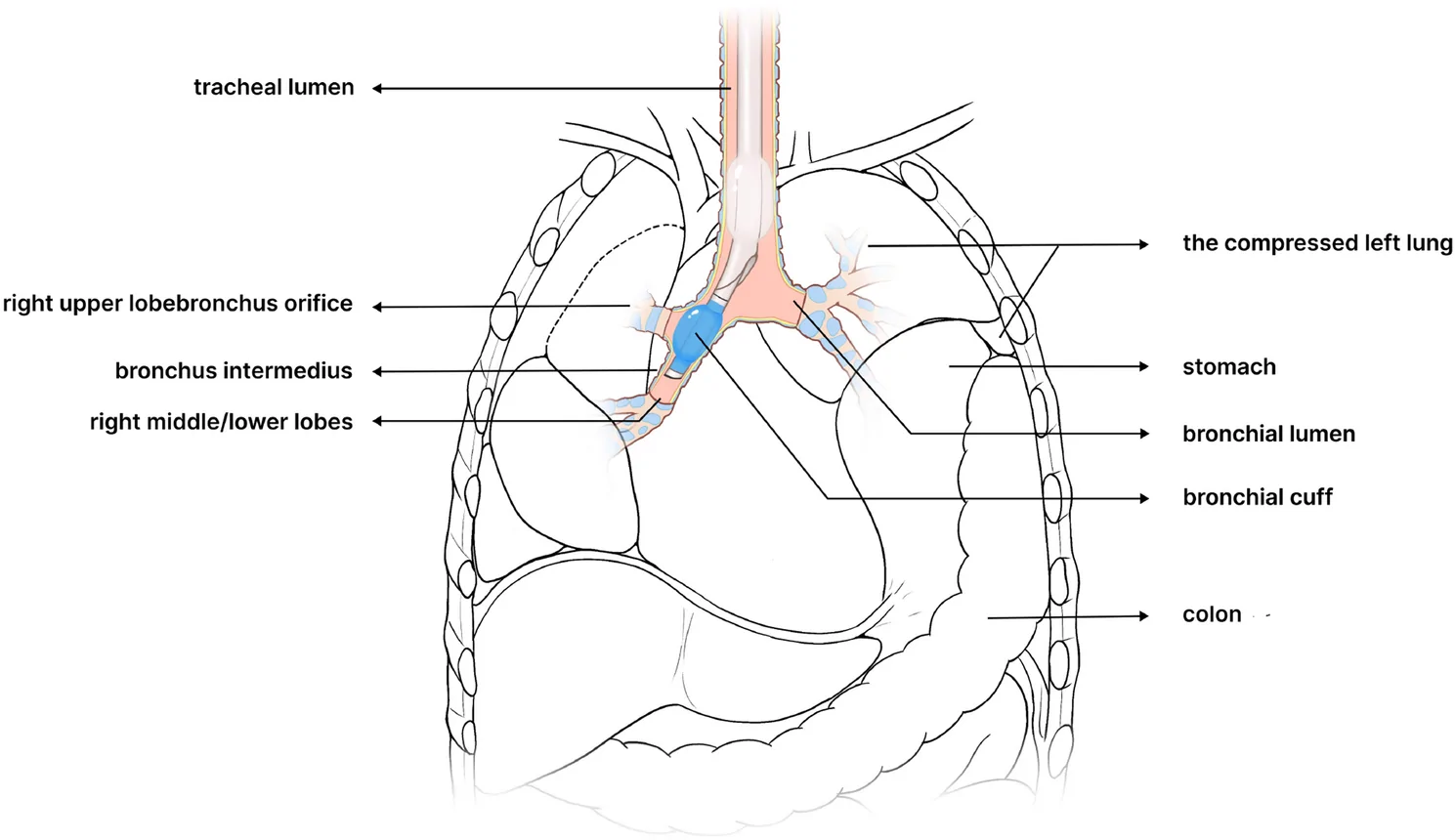
Schematic of selective right upper lobe isolation using a left-sided double-lumen tube. The bronchial cuff is inflated over the orifice of the right upper lobe bronchus to achieve selective occlusion, while the bronchial lumen ventilates the right middle and lower lobes and the tracheal lumen ventilates the compressed left lung. Herniated stomach and colon are shown occupying the left hemithorax

Preoperative CT measurements showed a right main bronchus diameter of 13 mm, a carina-to-right middle lobe (RML) bronchial orifice distance of 3.25 cm, and an RUL bronchial ostium diameter of 8 mm. Prior to intubation, the actual 32-Fr left-sided DLT (Shiley, Covidien) was physically measured, yielding a bronchial cuff-to-tip length (measured from the proximal cuff margin to the distal tip) of approximately 2.5 cm, a cuff length of approximately 12.0 mm, and an overall bronchial tube outer diameter of 10.7 mm. On the basis of these device-specific dimensions and the patient’s CT-measured anatomy, we considered that: (i) the right main bronchus diameter (13 mm) provided adequate clearance for tube passage; (ii) the RUL ostium was anatomically accessible for selective cuff occlusion under fiberoptic guidance. This tube was then inserted with the bevel facing left. After the bronchial cuff passed the vocal cords, the tube was rotated 90 degrees clockwise to direct the bronchial limb toward the right. It was then advanced into the right main bronchus under continuous fiberoptic bronchoscopy (FOB) guidance. The bronchial limb was positioned in the right bronchus intermedius, with the blue bronchial cuff placed just at the RUL orifice. To avoid obstructing the RML and right lower lobe (RLL) bronchi, the tip of the bronchial limb was kept proximal to the RML orifice; before cuff inflation, the bronchoscope was advanced through the bronchial lumen to directly visualize and confirm the patency of the RML and RLL bronchial openings beyond the tube tip. The bronchial cuff was then inflated with 2 mL of air using the minimal occlusive volume technique, and the cuff pressure was measured with a manometer and maintained at 20 cm H_2_O, which effectively occluded the RUL opening without encroaching on the lumen of the bronchus intermedius. The tracheal lumen opening was positioned approximately 2 cm above the carina, allowing free ventilation to the left lung. The final tube depth was 26 cm at the incisors, and this depth mark was recorded to facilitate intraoperative detection of displacement. The final position was confirmed by direct visualization: the tracheal lumen ventilated the left lung, and the bronchial lumen ventilated the right middle and lower lobes. A simplified schematic is shown in Fig. 2. No air leak was detected at a peak airway pressure of 30 cm H_2_O.

Because radiographic compression does not reliably predict intraoperative oxygenation during OLV, we first placed the patient in the left lateral decubitus position and confirmed the tube position by FOB, then attempted conventional left-lung OLV after positioning to objectively confirm intolerance. With the bronchial limb clamped (immediately restorable by unclamping), settings were fraction of inspired oxygen (FiO_2_) 100%, tidal volume 300–350 mL, respiratory rate 15 breaths/min, and positive end-expiratory pressure (PEEP) 5 cm H_2_O. The abort criterion was SpO_2_ <90% or a sustained downward trend. Within five minutes, SpO_2_ dropped from 100% to 89%, confirming intolerance. The bronchial clamp was immediately released, and ventilation was resumed through both lumens until SpO₂ returned to baseline. The tube position was rechecked by FOB to ensure no displacement before initiating the selective isolation strategy. The tracheal lumen ventilated the left lung, and the bronchial lumen ventilated the right middle and lower lobes.

**Fig. 3:**
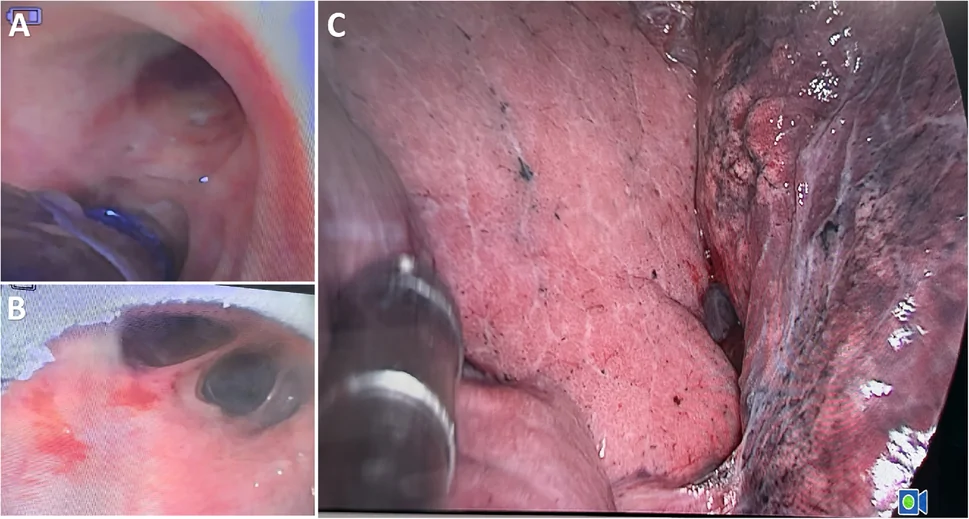
Intraoperative confirmation of selective right upper lobe isolation. **A** Fiberoptic bronchoscopic view through the tracheal lumen showing the carina and left main bronchus. **B** Fiberoptic bronchoscopic view showing the patent orifices of the right middle and lower lobe bronchial orifices distal to the cuff. **C** Thoracoscopic view during selective isolation: the ventilated right middle and lower lobes remained inflated and pink, whereas the non-ventilated right upper lobe appears collapsed and darker, with a visible demarcation along the oblique fissure

The RUL remained selectively collapsed because of bronchial cuff occlusion. Final ventilator settings were volume-controlled mode, tidal volume 300 mL (approximately 6 mL/kg ideal body weight), respiratory rate 14 breaths/min, PEEP 5 cm H_2_O, and FiO_2_ 0.6. To minimize the risk of tube displacement after positional changes, FOB verification was performed again after surgical draping. Subsequently, tube position was continuously monitored via capnography, peak airway pressure, and expired tidal volume trends. Additional FOB reconfirmations were scheduled before hilar dissection and RUL bronchial transection. Throughout the procedure, the patient’s head was kept in a neutral position and the tube was secured at the recorded insertion depth. No displacement of the tube or the bronchial cuff occurred during the 65-minute selective RUL isolation period, and no repositioning was required. Figure 3 demonstrates accurate tube placement and complete RUL collapse, which provided an adequate surgical field.

Before surgery, a predefined rescue protocol was established for the management of potential intraoperative emergencies. If SpO_2_ fell below 90%, surgical traction would be stopped and tube position would be assessed immediately using FOB. Bronchoscopic examination would be performed to identify potential causes, including distal tube migration resulting in RML obstruction, and proximal displacement resulting in RUL re-expansion. If oxygenation remained compromised, the bronchial cuff would be completely deflated to immediately restore ventilation through both lumens without exchanging the airway device. After stabilization, the tube would be repositioned under direct FOB guidance. If recurrent RUL re-expansion occurred while oxygenation remained adequate, an additional 0.2 to 0.5 mL of air would be added to the cuff under direct bronchoscopic visualization. Alternatively, the cuff would be partially deflated to the minimum sealing pressure, and bronchial stapling would be completed during a brief apneic pause. The rescue protocol was not activated because oxygenation and tube position remained stable. Intraoperative SpO_2_ remained between 97% and 100%, PaO_2_ ranged from 85 to 92 mmHg, and end-tidal carbon dioxide (EtCO_2_) was 36 to 42 mmHg. Hemodynamics remained stable throughout. The VATS right upper lobectomy was completed without difficulty. Total operative duration was 104 min, and the duration of selective RUL isolation was 65 min.

At the end of surgery, an intercostal nerve block was administered under direct thoracoscopic guidance. The patient regained consciousness smoothly and was extubated upon recovery of adequate muscle strength. She was transferred to the post-anesthesia care unit (PACU) for one hour of observation. Arterial blood gas analysis on supplemental oxygen (FiO_2_ 0.33) demonstrated mild hypercapnia (pH 7.30, PaCO_2_ 54.0 mmHg, PaO_2_ 99.8 mmHg), attributed to residual anesthetic effects and diminished ventilatory reserve. However, as she remained alert with adequate respiratory effort and stable oxygenation, noninvasive ventilation was deemed unnecessary. She returned to the ward with low-flow nasal oxygen (3 L/min), with successful weaning off supplemental oxygen by postoperative day 2.

Histopathological examination of the resected specimen confirmed Aspergillus infection within a cavitary lesion, surrounded by chronic inflammation and parenchymal fibrosis. Recovery was unremarkable, and she was discharged on postoperative day 6.

## Discussion

This case describes pre-planned selective RUL isolation using a left-sided DLT placed in the right bronchus intermedius during right upper lobectomy, preserving ventilation to the right middle and lower lobes and the compromised left lung. Surgical resection of localized pulmonary fungal disease is indicated for recurrent hemoptysis, antifungal failure, or suspected malignancy [8, 9], all three criteria were met. With the lesion confined to the RUL, we proceeded after confirming cardiopulmonary reserve. The estimated ppoFEV₁ of 52.0% and ppoDLCO of 41.2% fell within the ERS/ESTS intermediate-risk band (40–59%) [10], but this did not preclude surgery: controlling refractory hemoptysis and eradicating a destructive fungal cavity outweighed the moderate physiological risk. These estimates, derived from a non-validated segment-count approximation rather than perfusion-based methods, likely understated true reserve. Her clinical performance—ECOG 1, stair-climbing tolerance exceeding 4 metabolic equivalents, and resting SpO₂ of 95% on room air—was consistent with greater cardiopulmonary reserve. Her preserved functional capacity and compelling indication justified resection despite the intermediate-risk classification.

Conventional left-lung OLV, though technically straightforward, posed a high risk of intraoperative hypoxemia in this patient, as objectively demonstrated by the pre-incision desaturation trial. While extracorporeal membrane oxygenation (ECMO) is a well-established rescue modality for profound hypoxemia during one-lung ventilation, its prophylactic or emergent use in this elderly patient was considered unwarranted, given the availability of a less invasive airway strategy [11]. The potential risks of systemic anticoagulation, vascular complications, and resource utilization outweighed its perceived benefits in this patient [12]. A right-sided DLT ventilates rather than occludes the RUL via its side slot, and positioning is technically challenging [13]. A bronchial blocker (BB), though commonly used for selective lobar blockade in VATS [14], may interfere with RUL bronchial transection and carries a risk of dislodgment given the short RUL bronchus [15]. Additionally, it can be challenging to reposition rapidly if temporary conversion to complete right lung collapse is desired for hilar exposure. These considerations, combined with the larger and more secure bronchial cuff of the DLT, informed our device selection.

Our approach leveraged the left-sided DLT anatomical features to occlude the RUL bronchial orifice while preserving ventilation to the right middle and lower lobes (via the bronchial lumen) and the compromised left lung (via the tracheal lumen). Adapted placement of a left-sided DLT into the right bronchus intermedius to occlude the RUL orifice was first described by Alfery [16] to control RUL hemorrhage in a patient with structurally intact lungs. Omori et al. [3] later applied the same technique to seal an RUL bronchocutaneous fistula during positive pressure ventilation, again with both lungs anatomically normal. More recently, Gu et al. [17] reported its use for right anterior segmentectomy after left pneumonectomy, a scenario in which the contralateral lung was entirely absent. The present case extends this maneuver to elective right upper lobectomy for refractory pulmonary fungal infection in a patient whose contralateral lung was present but severely compromised. Unlike these prior reports, the current case integrates three elements into a unified clinical decision-making paradigm: (1) preoperative CT-based descriptive airway assessment to inform feasibility; (2) intraoperative physiological confirmation of OLV intolerance through a controlled desaturation trial; and (3) split-ventilation of both the ipsilateral dependent lobes and the compromised contralateral lung using standard equipment, thereby avoiding extracorporeal support. A pertinent consideration is whether transient conversion to complete right lung collapse, technically achievable by clamping the bronchial lumen, might be warranted to improve hilar exposure. In this patient, such a maneuver would need to be strictly time-limited and immediately reversible, given the documented physiological intolerance to isolated left-lung ventilation (SpO₂ declined to 89% within five minutes during the pre-incision trial).

The success of this maneuver depends on a narrow anatomical tolerance. In this patient, preoperative CT showed a right main bronchus of adequate caliber to accommodate the DLT. We emphasize, however, that these CT measurements serve only as a preliminary guide to device-patient compatibility; definitive safe placement relies on direct fiberoptic bronchoscopic confirmation of preserved RML and RLL patency. We therefore recommend a stringent protocol of repeated FOB verification at critical intraoperative time points for safe execution. On the basis of our experience, we suggest that the minimum anatomical considerations for attempting this technique include: (i) a right main bronchus diameter sufficient to accommodate the DLT outer profile, and (ii) an airway configuration that permits the bronchial cuff to seat at the RUL ostium while the tip remains proximal to the RML orifice, as confirmed by FOB. Patients with less favorable airway dimensions, including a shorter distance between the RUL and RML orifices or narrower bronchi, may not be ideal candidates and require careful individualized assessment.

Given the narrow anatomical tolerance, distal migration could obstruct the dominant right middle and lower lobes, while proximal migration could re-inflate the RUL. Surgical manipulation also risks kinking or traction. We mitigated these risks by documenting tube depth, neutral head positioning, continuous capnography and pressure monitoring, and repeat FOB verification at key stages. In addition, a predefined intraoperative emergency rescue workflow was established to enable prompt bronchoscopic assessment, immediate restoration of ventilation, and tube repositioning in the event of hypoxemia or tube displacement. These preventive and rescue measures supported the safe implementation of this technique.

This report has limitations. First, the narrow anatomical tolerance mandates precise FOB guidance, patients with smaller bronchi are unsuitable. Second, selective collapse may limit hilar exposure, and transient conversion to conventional OLV, though theoretically possible, must be strictly time-limited given this patient’s poor tolerance. Third, preoperative microbiological confirmation was incomplete. Fourth, quantitative perfusion scintigraphy was not performed; predicted postoperative values were therefore estimated by a non-validated segment-count method that assumes radiographic compression corresponds proportionally to regional functional loss, an assumption that may not reflect actual physiology and introduces uncertainty into risk stratification. Finally, the airway maneuver itself is not novel, the value lies in its pre-planned indication, objective intraoperative validation, and split-ventilation strategy for partially impaired contralateral lungs.

## Conclusions

For patients with severe contralateral lung dysfunction undergoing right upper lobectomy, selective RUL isolation using a left-sided DLT placed in the right bronchus intermedius is feasible when CT shows adequate right main bronchus caliber and fiberoptic bronchoscopy directly verifies safe cuff placement at the RUL orifice, a tip position proximal to the RML orifice, and preserved RML/RLL patency. This technique requires a predefined rescue protocol and should be reserved for carefully selected patients, not as a routine substitute for standard OLV.

## Data Availability

All data generated or analyzed during this study are included in this published article. The datasets used and/or analyzed during the current study are available from the corresponding author on reasonable request.

## Abbreviations

ABG: Arterial Blood Gas
BB: Bronchial Blocker
CT: Computed Tomography
DLCO: Diffusing Capacity of the Lung for Carbon Monoxide
DLT: Double-Lumen Tube
ECMO: Extracorporeal Membrane Oxygenation
ECOG: Eastern Cooperative Oncology Group Performance Status
EtCO_2_: End-Tidal Carbon Dioxide
FEV₁: Forced Expiratory Volume in 1 s
FiO_2_: Fraction of Inspired Oxygen
FOB: Fiberoptic Bronchoscopy
FVC: Forced Vital Capacity
OLV: One-Lung Ventilation
PACU: Post-Anesthesia Care Unit
PEEP: Positive End-Expiratory Pressure
ppoDLCO: Predicted postoperative DLCO
ppoFEV₁: Predicted postoperative FEV₁
RLL: Right Lower Lobe
RML: Right Middle Lobe
RUL: Right Upper Lobe
VATS: Video-Assisted Thoracoscopic Surgery
